# Reproducibility of brain metabolite concentration measurements in lesion free white matter at 1.5 T

**DOI:** 10.1186/s12880-015-0085-9

**Published:** 2015-09-29

**Authors:** Martin H J Busch, Wolfgang Vollmann, Serban Mateiescu, Manuel Stolze, Martin Deli, Marietta Garmer, Dietrich H W Grönemeyer

**Affiliations:** Grönemeyer Institut für Mikrotherapie, Universitätsstraße 142, D-44799 Bochum, Germany; Beuth Hochschule für Technik Berlin, Luxemburger Straße 10, D-13353 Berlin, Germany; Ruhr Universität Bochum, Universitätsstraße 150, D-44801 Bochum, Germany; Amedo Smart Tracking Solutions, Universitätsstraße 142, D-44799 Bochum, Germany

## Abstract

**Background:**

Post processing for brain spectra has a great influence on the fit quality of individual spectra, as well as on the reproducibility of results from comparable spectra. This investigation used pairs of spectra, identical in system parameters, position and time assumed to differ only in noise. The metabolite amplitudes of fitted time domain spectroscopic data were tested on reproducibility for the main brain metabolites.

**Methods:**

Proton spectra of white matter brain tissue were acquired with a short spin echo time of 30 ms and a moderate repetition time of 1500 ms at 1.5 T. The pairs were investigated with one time domain post-processing algorithm using different parameters. The number of metabolites, the use of prior knowledge, base line parameters and common or individual damping were varied to evaluate the best reproducibility.

**Results:**

The protocols with most reproducible amplitudes for N-acetylaspartate, creatine, choline, myo-inositol and the combined Glx line of glutamate and glutamine in lesion free white matter have the following common features: common damping of the main metabolites, a baseline using only the points of the first 10 ms, no additional lipid/macromolecule lines and Glx is taken as the sum of separately fitted glutamate and glutamine. This parameter set is different to the one delivering the best individual fit results.

**Discussion:**

All spectra were acquired in “lesion free” (no lesion signs found in MR imaging) white matter. Spectra of brain lesions, for example tumors, can be drastically different. Thus the results are limited to lesion free brain tissue. Nevertheless the application to studies is broad, because small alterations in brain biochemistry of lesion free areas had been detected nearby tumors, in patients with multiple sclerosis, drug abuse or psychiatric disorders.

**Conclusion:**

Main metabolite amplitudes inside healthy brain can be quantified with a normalized root mean square deviation around 5 % using CH_3_ of creatine as reference. Only the reproducibility of myo-inositol is roughly twice as bad. The reproducibility should be similar using other references like internal or external water for an absolute concentration evaluation and are not influenced by relaxation corrections with literature values.

## Background

Nuclear magnetic resonance spectroscopy has developed over the last decades as one of the exciting fields in medical physics for the non-invasive measurement of molecular concentrations inside the body [1–7]. In principle the method is widely available at magnetic resonance imaging (MRI) units with field strengths above 1 T but its application is restricted for two main reasons. Although data acquisition can be performed automatically for optimal results an experienced operator is necessary. Additionally no standard post processing of the acquired data exists [8]. This investigation tries to enhance the post processing towards reproducible results by varying the post processing parameters for identically acquired spectra pairs.

Biochemical concentrations inside specific body regions can be evaluated by fitting magnetic resonance spectroscopic data using model signals of the metabolites to be quantified. The fit algorithm detects metabolites with a fair signal to noise ratio and sufficient long spin-spin relaxation time T_2_ in relation to the echo time TE. Metabolites with short T_2_’s (broad lines) are hardly separable and combined as baseline.

Metabolite model signals in time domain have the following parameters adjustable by the fit algorithm: amplitude (corresponding to the area under a line in frequency domain data), frequency (ppm position), damping (line width) and phase (influencing the line shape in frequency domain) [9–11]. The adoption of “prior knowledge” can reduce the number of adjustable parameters and/or restrict them to a certain region. For example: the metabolite frequency is fixed to another metabolite or limited to a region around the theoretical value; the metabolites completely or partly have a common damping or phase.

Fit procedures for spectral data adjust the parameters for the specified metabolites to minimize *χ*^2^ respecting all prior knowledge restrictions. Usually, *χ*^2^ in time domain is defined as the sum Σ_i_ |Δ_i_|^2^, where Δ_i_ is the difference between measured value and fit value for the data point sampled at time t_i_. If the assumed errors σ_i_ of the measured values are not identical for all i, *χ*^2^ is taken as Σ_i_ |Δ_i_/σ_i_|^2^ to weight data points according to the error σ_i_ in measurement i. For the ideal case, fitting of all metabolites with appropriate parameters should result in a difference between fit and the acquired signal around zero containing the pure noise signal. This ideal case is not achievable, because signals can be masked by noise, they can overlay each other or signals with low T_2_ (broad lines) are not completely represented by the base line. But it is evident that a flat difference line with a low *χ*^2^ can be easily achieved using a large parameter set (*i. e.* using many metabolites with all four parameters of each metabolite freely adjustable).

Until today no commonly accepted post processing exists to quantify concentrations. Firstly, frequency [12] or time domain models [10, 11] can be used for the appropriate data. Secondly, a decision is necessary on the metabolites taken into account and on the restrictions of each metabolite’s post processing parameters by the use of prior knowledge.

For a series of acquisitions the fit quality can be rated by the mean of all *χ*^2^. Using different metabolite sets with varying post-processing parameters can detect a minimal mean *χ*^2^. A large set of metabolites with unrestricted parameters decreases each *χ*^2^ and hence also the mean of all χ^2^’s. But such a parameter setting not necessarily is the one evaluating metabolite concentrations with the best reproducibility. A post processing with a good reproducibility should compute nearly identical metabolite concentrations for data known to have the same metabolite basis.

As approach for the preparation of *in vivo* data, differing only in noise, ideally a complete acquisition with 2 N additions is divided in two acquisitions with N additions. The two spectra for comparison build a pair consisting of the sum of all even and the sum of all odd acquired signals. The spectra of this procedure are as reproducible as possible for *in vivo* acquisitions. The MR-system software did not allow adding even and odd excitations to summed signals. Instead the two spectra of a pair were acquired directly consecutively with the same system parameters at an identical position and time point (identical in time with respect to physiological concentration changes). As long as the investigated subject did not move within the two acquisitions this should match the best achievable reproducibility for *in vivo* spectroscopy. For the comparison it is assumed to have a pair of two spectra formed by an identical metabolite basis overlaid with different noise. The post processing should evaluate similar results for the main (clearly visible) metabolites.

This investigation concentrates on one post processing software package metabolite report [9, 13, 14], mostly identical to the new product software “syngo via” from Siemens Healthcare (Erlangen, Germany) neglecting all aspects and discussions on acquisition schemes or comparisons to other post processing algorithms.

The purpose of this paper is to achieve a good reproducibility of the major metabolite amplitudes from the acquired pairs of *in vivo* spectra by adjusting the post processing fit parameters. These amplitudes are the basis for all relative or absolute concentration calculations.

## Methods

### Participants

This study was retrospective with the data of a master thesis in Biology at the Ruhr University of Bochum (RUB). All participants had given written informed consent. The local ethics committee of the RUB approved the study and all data evaluations.

Fifty-four spectra pairs were acquired, 28 pairs from six females and 26 pairs from five males (Table 1). None of the healthy subjects showed any evidence of a brain lesion according to the acquired T_2_-weighted sequences. All subjects were of similar age. Gender and age were similarly distributed on the number of subjects as well as on the number of spectra acquisitions (Table 1).

**Table 1:** Subject demographics

Healthy subject no	Gender	Age male	Age female	No of investiga-tions male (spectrum pairs)	No of investiga-tions female (spectrum pairs)
1	f		23.9		4 (8)
2	f		24.9		4 (8)
3	m	24.1		5 (10)	
4	f		22.6		2 (4)
5	m	21.2		2 (4)	
6	f		22.7		2 (4)
7	m	24.1		2 (4)	
8	m	25.9		2 (4)	
9	m	23.6		2 (4)	
10	f		20.5		1 (2)
11	f		27.7		1 (2)
Mean		23.8	23.7	2.6 (5.2)	2.3 (4.6)
sd		1.7	2.4	1.3 (2.7)	1.4 (2.7)
Mean		23.7		2.5 (4.9)	
sd		2.0		1.3 (2.6)	

### Spectra acquisition

Subjects were positioned head first, supine with their head slightly fixed inside the coil. All measurements were carried out on a Magnetom Espree (horizontal 1.5 T field, wide bore, Siemens Healthcare, Erlangen, Germany). The signal was received with a 12-channel head matrix coil and the circularly polarized body coil was used for excitation. Firstly, for exclusion of any brain lesion as well as for spectroscopy voxel placement, T_2_ weighted axial “Turbo Spin Echo” images of the entire brain were acquired followed by two (coronal and sagittal) “True Fisp” (true fast imaging with steady state precession) sequences or a sagittal “True Fisp” and a coronal “Tirm” (turbo inversion recovery magnitude) sequence for voxel placement (sequence parameters in Table 2).

**Table 2:** Imaging sequence parameters

Sequence name	Orientation	TR [ms]	TE [ms]	FA [°] TI [ms]	TA [s]	FOV [mm x mm]	Matrix [points]	Slice thick. [mm]
TSE	axial	5950	105		55	180 × 240	276 × 512	5
True Fisp	cor/sag	4,8	2,4	70°	29	180 × 240 240 × 240	192 × 256	5
Tirm	cor	8510	138	2500 ms	60	180 × 240	320 × 512	6

*TR* repetition time, *TE* echo time, *TI* inversion time, *FA* flip angle, *TA* acquisition time, *FOV* field of view

**Fig. 1:**
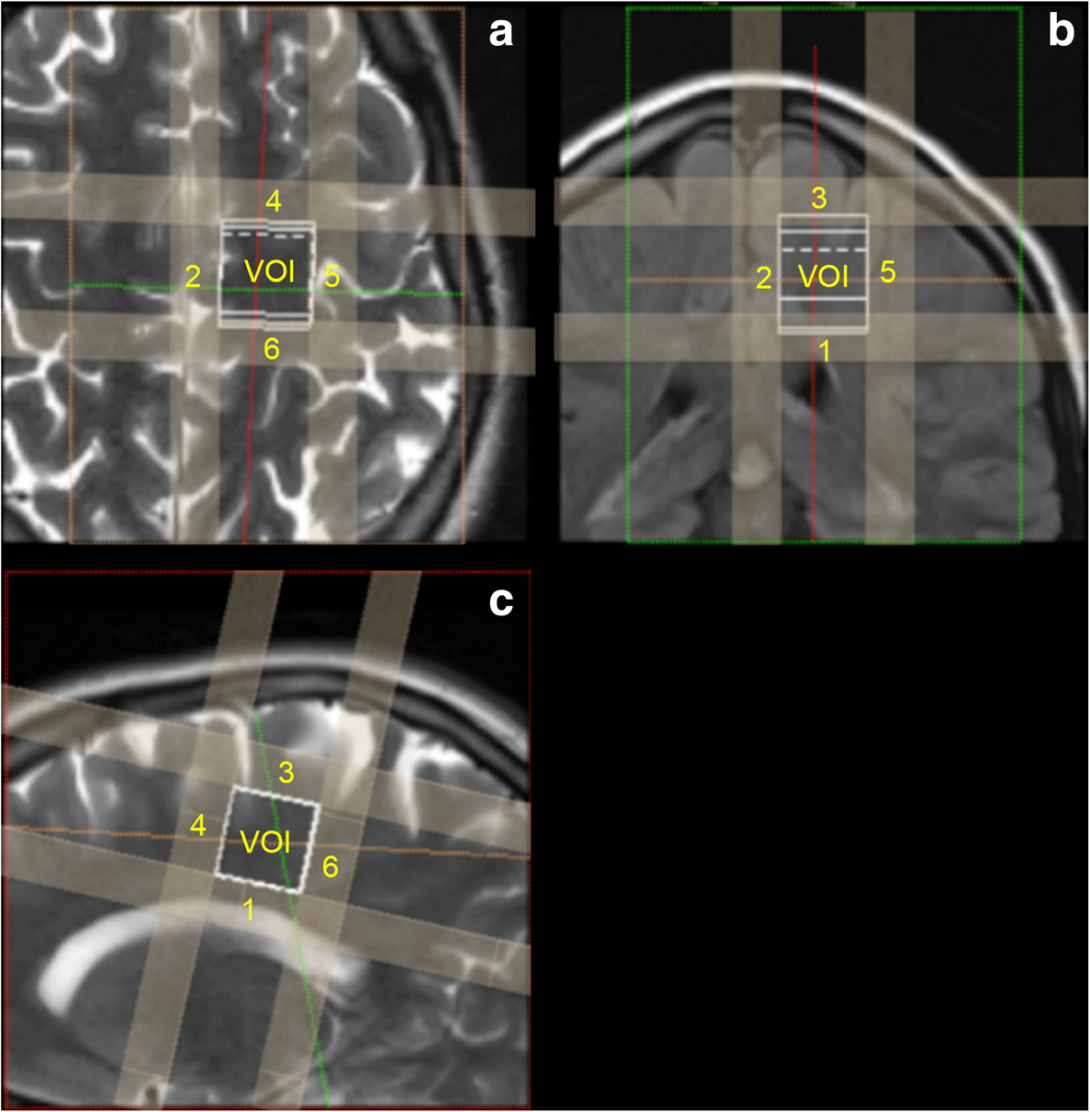
Example of voxel position (VOI) in axial (**a**), coronal (**b**) and sagittal (**c**) images, as well as the saturation regions (numbered from 1 to 6)

Two pairs of “point resolved single voxel spectra” (PRESS) were acquired with identical system parameters and localized at an identical position on each brain hemisphere for each subject. Always cubic voxels of 2 cm × 2 cm × 2 cm were selected and placed on the left and right brain hemisphere central in anterior posterior direction above the ventricle (marked in Fig. 1 as VOI). Position and orientation were adjusted to contain white matter as much as possible. Voxels were placed within ±5 cm relative to the central z-axis position (center of the magnet in head feet direction). Six saturation pulses were positioned along each plane of the cube (Fig. 1) for outer volume suppression (marked in Fig. 1 as numbered bars surrounding the VOI). The most effective one was oriented towards the ventricle (bar 1 in Fig. 1) and next effective one towards the midline of the cerebrum (bar 2 in Fig. 1). Prior to paired spectra acquisitions on each side, system parameters were automatically adjusted and the magnetic field inside the voxel was homogenized by an automatic shim. Additionally an experienced operator (MB or SM) optimized shim and water suppression.

One thousand twenty four data points were sampled in 512 ms in a two-fold oversampling technique. In the thesis of MS the short sampling window of 512 ms enables faster repetition times TR for measuring spin–lattice relaxation times T_1_. The echo time TE was short (30 ms) and the repetition time TR moderate (1500 ms). The two spectra of a pair were acquired directly consecutive using 128 additions for each spectrum. For signal evaluation the signal of all 12 channels of the head coil was used. Total acquisition time on one side was 6:36 min (2 × 3:18 min) preceded by the system parameter adjustment time of 7 to 10 min. Total MR time for each subject was about 30 to 40 min for one session.

### Post-processing

The post-processing software “metabolite report” was a work in progress package from Siemens Healthcare. It is the precursor of the currently available commercial spectroscopy software within the new product “syngo via” and nearly identical to this package (personal communication with Elisabeth Weiland [15], see acknowledgement). “*Automated post processing of this software package consists of the following steps: 1] identification of the prominent metabolites by cross-correlation to a database (i.e., N-acetylaspartate (Naa), choline (Cho), creatine (Cre), myoinositol (Myo) for short echo brain data), 2] determination of the B*_*0*_*shift and starting values for the fit parameters, 3] residual water removal, and 4] fit of the spectroscopic data. The fit is based on a basis set of metabolic model signals, which are simulated with the use of literature values for chemical shifts and coupling constants. In addition, truncating and remodeling of the first data points handle baseline artifacts*” [9, 15].

For the analysis of the short TE data of this study, the following metabolites were included in the fit (major metabolites are marked bold):

- Creatine with separately fitted CH_2_ and CH_3_ groups (Cre2 and **Cre3**)
- N-Acetylaspartat (**Naa**)
- Choline (**Cho**)
- Myo-inositol (**Myo**)
- **Glx** as a combined Glu/Gln model or separately fitted Glutamate (Glu) and Glutamine (Gln) with the sum of both amplitudes as **Glx** value (in case of a not found Gln, Glu alone was used as Glx value)
- Two lipid/macromolecule lines around 0.9 and 1.3 ppm in some cases (Lipid_1, Lipid_2)
- GABA (Gamma Amino Butter Acid) in a few cases

**Fig. 2:**
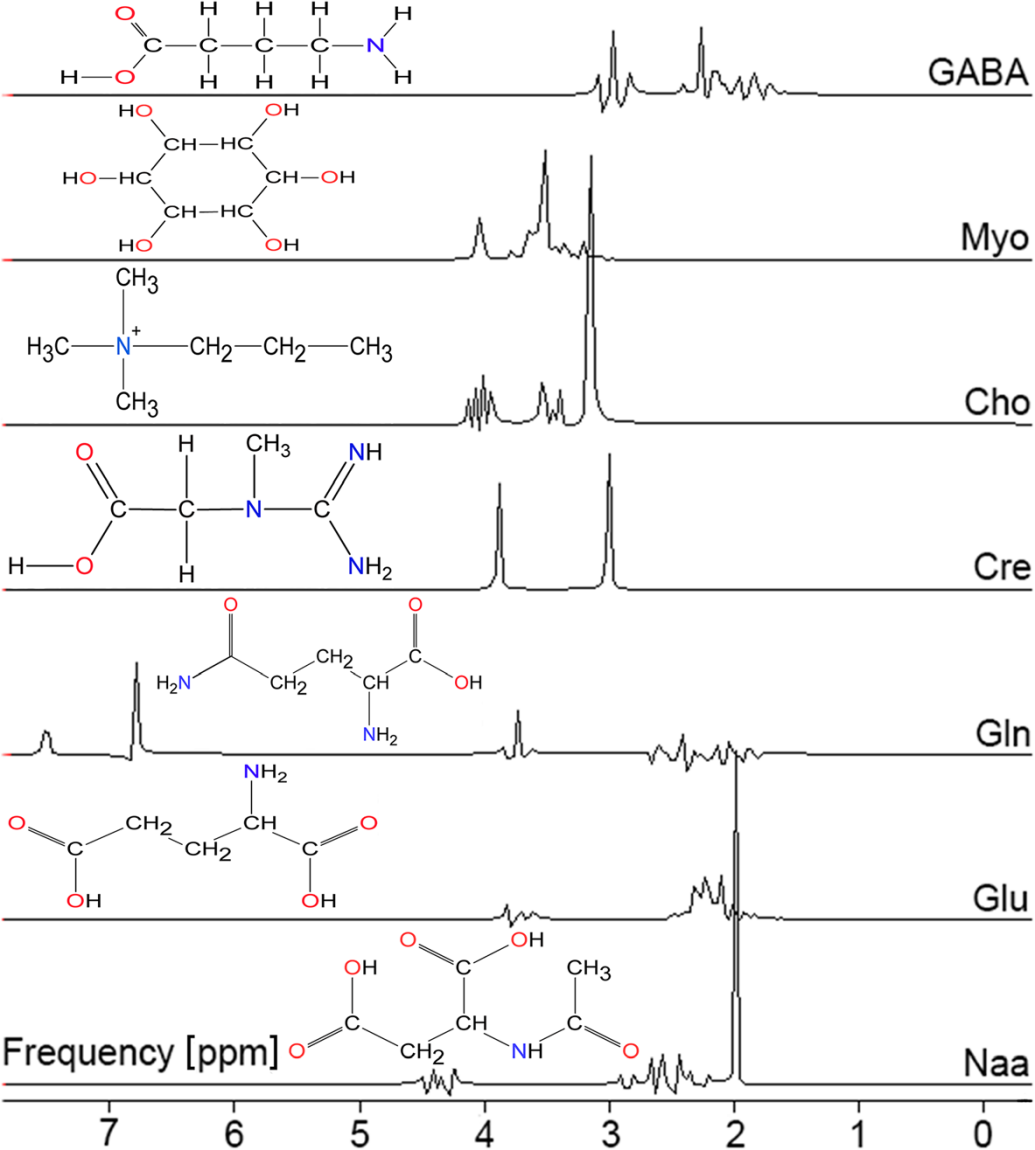
Model metabolites in frequency domain for a 30 ms PRESS sequence calculated with NMR-Scope of jMRUI using a damping of 2 Hz [10, 11]. (Chemical structures build with free Chemscetch from ACD Labs, Toronto, Canada)

Metabolites were modeled as Gauss signals. Only the lipids were parameterized as Lorentz singlets with individual damping (line width) as recommended for short echo time acquisitions at 1.5 T [16, 17]. All model signals were filtered by a pass band from 0 to 4.2 ppm to exclude frequencies outside the area of interest (see model files in Fig. 2). This pass band filter influences the amplitude for relative or absolute concentration calculations by a constant factor respected within the software metabolite record. For pure reproducibility investigations a constant factor is not relevant.

For each metabolite the amplitude, the frequency and the damping (line width) are parameters obtained from the fit procedure. It is possible to use an individual phase of each model signal as fit parameter, but as commonly accepted a joint phase is used for all metabolites. The fitted metabolites had to be above the noise level in frequency domain by default factor of eight. Otherwise the metabolite is omitted and the fit is restarted without this metabolite (personal communication with EW). In this study the default factor was never changed. The software marks metabolites chosen for the fit but omitted, because it is too small in signal to noise. For reproducibility evaluation a decision is necessary how to respect pairs with a one signal above and the other below the noise level. In our investigation the not found signal was set to zero. In case of both signals below the noise level this pair was not respected.

A biochemical concentration is calculated with the fitted amplitude of a metabolite. Therefore, the post processing parameters should be optimized to deliver reliable and most reproducible amplitudes (proportional to metabolite concentration) for acquisitions assumed to differ only in noise. The frequency of a metabolite line was adjustable by the fit algorithm within boundaries to the theoretical value or it could be referenced to a fixed distance to another metabolite, which frequency can be more easily detected. If the frequency was not fixed to that of another metabolite the bounds were set to the theoretical value ± 0.1 ppm for all metabolites except the lipids (±0.2 ppm). The damping could be fitted individually for each metabolite or one or more groups of metabolites could have a common damping. The damping for single metabolites or a group of metabolites was restricted to values between 1 and 10 Hz except for lipids (1 to 25 Hz, see below).

The separate fit of the CH_3_ and CH_2_ creatine lines was necessary, because the residual water suppression diminishes the amplitude of the CH_2_ group disturbing the theoretical ratio of 3/2. The water removal consists of an iterative low pass filter around the frequency at 4.7 ppm with a fixed number of 22 iterations. At 1.5 T an accurate separation of Glu and Gln is difficult. Therefore Glx as a combined model with a fixed concentration relation between both or the sum of separately fitted Glu and Gln was evaluated as one of the main metabolites. The baseline was varied using 20 or 50 data points corresponding to 10 or 25 ms, respectively. The two “dummy lipids” were introduced as additional lines to model the base line solely below 1.8 ppm. These lipid lines incorporate macromolecules.

For detecting the most reproducible results using pairs of consecutive acquisitions with identical system parameters, no relaxation corrections had to be applied to the fitted amplitudes. It is sufficient to examine the unchanged amplitudes from which relative or absolute concentrations can be calculated. The error most likely increases (reproducibility decreases) with corrections and/or evaluation of absolute concentrations [mmol/l] or relative concentrations referenced to another metabolite. Both relaxation corrections as well as evaluation of concentrations involve measured or assumed values increasing the concentration errors.

### Reproducibility calculations

Reproducibility is calculated with two directly consecutive acquired spectra (a pair) on one side of the brain. They are identical concerning the biochemical metabolite basis including the ones with a short T_2_ simulated as base line and they coincide in system parameter settings. For concentration independent quantification of the reproducibility the normalized squared difference of a pair is calculated. The possible range of the normalized difference is between −2 and 2. Case of “±2” consists of a zero and a non-zero value, which means the non-zero value is 200 % of the mean value. Typical values are much smaller than 1 for each main metabolite. The root of the mean value of all acquired pairs defines the normalized root mean square deviation, which simply is designated as reproducibility number rn (Eq. 1).

1 $$ rn(met)=\sqrt{\frac{1}{N}{\displaystyle {\sum}_{i=1}^N{\left(\frac{\left( am{p}_{i_2}(met)- am{p}_{i_1}(met)\right)}{\left( am{p}_{i_2}(met)+ am{p}_{i_1}(met)/2\right)}\right)}^2}} $$

rn(met): reproducibility number; normalized root mean square deviation for metabolite “met”
N: number of acquisition pairs
amp_ij_(met): amplitude of metabolite met for acquisition pair with index i. The index within a measurement pair is j = 1, 2.

The “reproducibility number” rn(met) describes the mean relative (percentage) deviation between N measurement pairs for metabolite met. So a low rn(met) value means a good reproducibility of metabolite met.

For the calculation of rn no reference is necessary to exclude different system settings, because the compared values of a pair were acquired with identical parameters. Normalization to the mean value was used to ease the reproducibility comparison between different metabolites.

Firstly the reproducibility is tested with only one spectrum pair of the eleven subjects. The first acquisition pair on the left-brain hemisphere is evaluated using the parameters specified in table 3 for 25 different protocols. As measure of reproducibility for all metabolites the added rn percentage value of Naa, Cre3, Cho, Myo and Glx was used (Table 3). Other sums of rn with less metabolites were also calculated for overall reproducibility rating. Post processings with a low sum (marked underlined and italic Table 3) were evaluated for all 54 pairs (Table 4).

**Table 3:** Description of tested protocols

Protocol no	Glx	Glu/Gln	GABA	Li’s	Base	com. dam.	full eval.
0	free	–	–	–	25 ms	–	–
1	free	–	free	free	25 ms	–	–
1a	free	–	–	free	25 ms	–	–
2	free	–	–	–	10 ms	–	–
3	free	–	free	free	10 ms	–	–
3a	free	–	–	free	10 ms	–	–
4	–	free	–	–	25 ms	–	–
5	–	free	free	free	25 ms	–	–
5a	–	free	–	free	25 ms	–	–
6	–	free	–	–	10 ms	–	+
7	–	free	free	free	10 ms	–	–
7a	–	free	–	free	10 ms	–	–
8	–	Naa	–	–	25 ms	–	–
9a	–	Naa	–	free	25 ms	–	–
10	–	Naa	–	–	10 ms	–	+
11a	–	Naa	–	free	10 ms	–	–
12	–	free	–	–	25 ms	+	+
13a	–	free	–	free	25 ms	+	+
14	–	free	–	–	10 ms	+	+
15a	–	free	–	free	10 ms	+	–
16	–	Naa	–	–	25 ms	+	–
17a	–	Naa	–	free	25 ms	+	–
18	–	Naa	–	–	10 ms	+	+
19a	–	Naa	–	free	10 ms	+	+
20	–	Naa	–	free	10 ms	+ Myo free	–

Amplitude and frequency of Naa, Cre3, Cre2, Cho and Myo are always fit parameters. The damping for each metabolite is independent in case of a “–” in column “common damping” and equal for all metabolites except the lipids and Cre2 in case of a “+”. The damping of these metabolites can be adjusted between 1 and 10 Hz. The damping of lipids is always independent with range from 1 to 25 Hz. “full evaluation” means all 54 pairs of spectra were used for rn evaluation, whereas in other cases only one spectrum of each subject (11 examples, left brain hemisphere of first investigation) was used for an estimation of rn. Naa means the frequency of Glu/Gln is fixed relative to the detected frequency of Naa. The numbering follows the historical order of evaluation and therefore needs in some cases an index “a” for later evaluated protocols

**Table 4:** Reproducibility results using 11 left brain pairs of spectra from eleven subjects

	Glx all metabolites with free parameter						Glu. Gln ref Naa			
Protocol	0	1	1a	2	3	3a	8	09a	10	11a
Naa	3.1	8.8	2.9	4.4	16.4	5.5	2.9	2.9	3.3	3.2
Cre3	5.4	6.0	5.5	5.3	7.6	5.9	5.4	5.5	5.6	5.5
Cho	4.5	6.1	4.7	4.6	11.0	7.6	4.4	4.4	4.7	7.2
Myo	8.3	9.8	9.7	17.4	52.6	46.5	6.8	6.4	11.3	33.5
Glx	153.1	167.5	159.0	82.0	127.1	115.6	13.6	12.8	5.2	**5.0**
all	174.4	198.3	181.7	113.7	214.7	181.1	33.2	31.9	*30.1*	54.4
Naa. Cre3. Cho	13.0	21.0	13.1	14.2	35.0	19.0	12.8	12.7	13.6	15.9
all–Myo	166.1	188.4	172.0	96.3	162.1	134.6	26.4	25.5	18.8	20.9
all–Glx	21.3	30.8	22.7	31.6	87.6	65.6	19.6	19.1	24.9	49.4
Mean *χ* ^2^	3819	3617	3839	5574	4731	5120	3742	3765	5040	4941
	Glu. Gln all metab. with free parameter						Glu. Gln. all free + co. dam.			
Protocol	4	5	5a	6	7	7a	12	13a	14	15a
Naa	3.3	12.0	3.4	4.7	9.8	7.7	2.9	2.8	3.2	5.2
Cre3	5.3	4.8	5.3	5.3	5.9	5.1	**4.3**	**4.3**	5.1	5.5
Cho	4.2	5.4	4.1	4.1	8.6	5.1	3.7	3.6	**3.3**	5.5
Myo	13.8	15.9	12.1	6.4	39.9	31.1	6.3	**5.1**	8.7	17.3
Glx	25.9	25.0	22.8	**5.0**	14.4	7.5	13.1	12.1	6.1	6.9
all	52.5	63.0	47.8	*25.4*	78.6	56.4	*30.3*	*27.9*	*26.3*	40.3
Naa. Cre3. Cho	12.8	22.1	12.8	14.0	24.3	17.8	10.9	10.7	11.5	16.2
all–Myo	38.7	47.1	35.6	19.1	38.7	25.3	24.0	22.8	17.6	23.1
all–Glx	26.6	38.0	24.9	20.4	64.2	48.9	17.2	15.8	20.2	33.5
Mean *χ* ^2^	3651	3475	3751	4773	4399	4667	3757	3786	5041	4891
	Glu. Gln ref Naa + common damping									
Protocol	16	17a	18	19a	20					
Naa	2.8	**2.7**	3.2	3.1	3.0					
Cre3	4.4	4.4	5.2	4.9	4.9					
Cho	3.5	3.5	**3.3**	3.4	4.7					
Myo	5.2	5.2	10.8	11.1	6.9					
Glx	22.4	23.2	6.2	5.8	29.9					
all	38.3	39.0	*28.7*	*28.4*	49.4					
Naa. Cre3. Cho	10.7	10.7	11.7	11.4	12.6					
all–Myo	33.1	33.9	17.9	17.2	42.5					
all–Glx	15.9	15.8	22.5	22.6	19.6					
Mean *χ* ^2^	3856	3886	5237	5138	5046					

The minimal values for each of the five main metabolites are marked bold. “Sums” of all five metabolite rn’s below 31 are marked underlined italic. These eight protocols are investigated for all 54 pairs of spectra. The mean *χ* ^2^ value is calculated from the 22 Siemens post processing *χ* ^2^ of the fit procedures necessary to calculate the rn of 11 pairs. Note: all values in percent except for mean *χ* ^2^

Additionally the metabolite amplitudes for the protocols with the best reproducibility were referenced to the amplitude of Cre3 (amp_ref_(met) = amp(met)/amp(Cre3)) to check the influence on this step to the reproducibility.

In principal an unlimited number of different post-processing protocols are possible. Hence in this investigation the protocols were varied to answer the following questions.

Is the reproducibility increased or decreased by the use of:

1. additional low signal to noise metabolites beside the major metabolites?
  To answer this question as example GABA was used with peaks in between 2 and 3 ppm (Fig. 3a). N-Acetyl Aspartyl Glutamate (NaaG) was also investigated, but in this case the Naa evaluation was distorted too much even using the sum of Naa and NaaG (Fig. 3c) and therefor was not further respected.

**Fig. 3:**
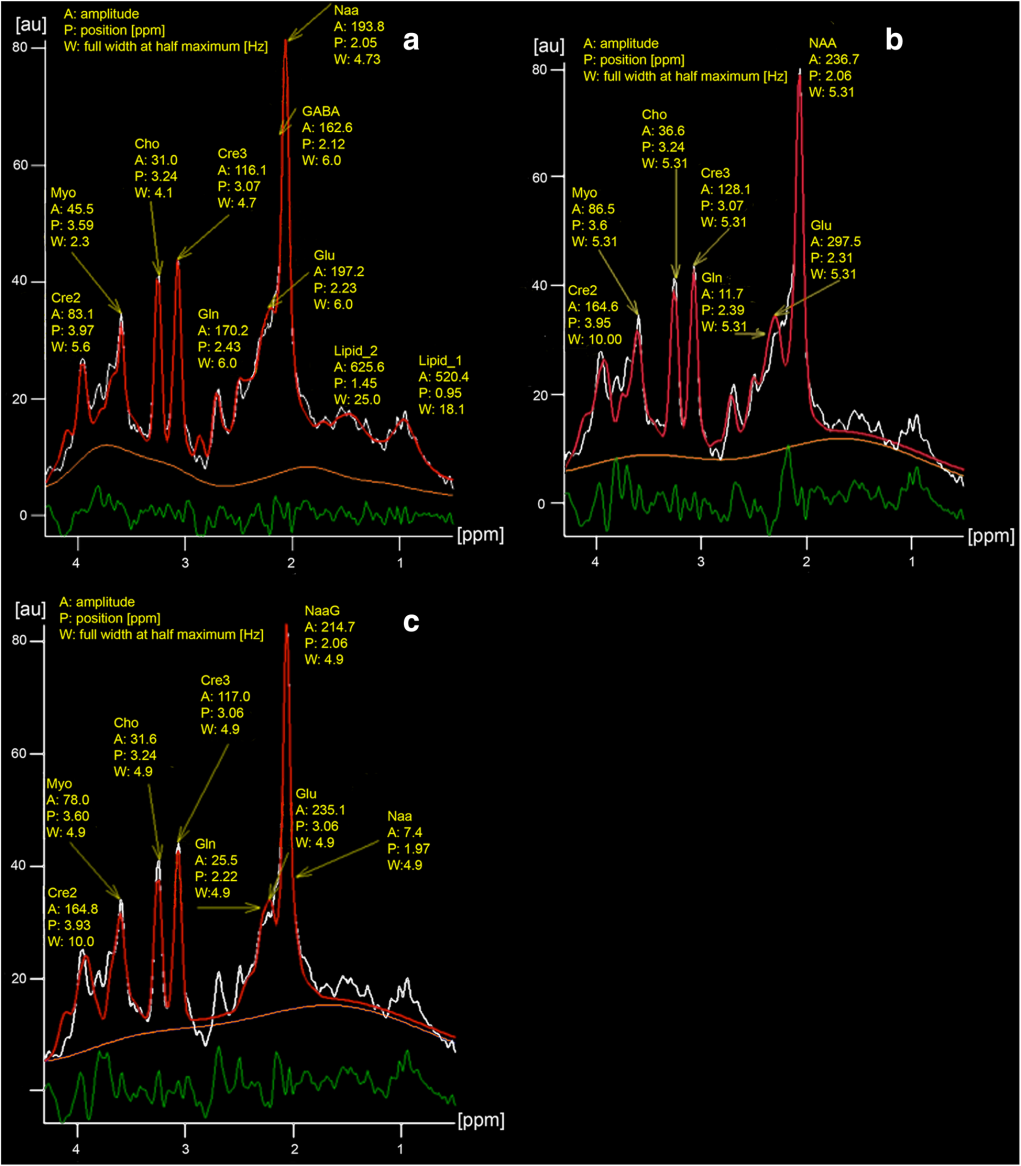
Fit (*red*) of the same data (*white*) calculated with protocol 5 resulting in a “flat” (**a**) and with protocol 18 with a “less flat” difference line (*green*) (**b**). The baseline is orange. *χ* ^2^ calculated by the post processing software was 3348 (**a**) and 5122 (**b**), respectively and is a little less than the mean values of Table 3. Image (**c**) shows the distorted Naa evaluation with NaaG as additional metabolite. *A*:, *P*: and *W*: are the amplitude of a metabolite, its frequency in ppm and its line width in Hz. Note: the lipid amplitudes inside healthy brain tissue are very small compared to that of tumors. In most cases, depending on the protocol, no or only one lipid line was detected. This is also documented by both protocols with the best reproducibility (protocol 14 and 18), which did not use lipid models
2. separately fitted glutamate and glutamine values instead of a predefined Glx model?
3. a flat or a more curved baseline using the first 10 or 25 ms for the base line calculation?
4. an independent damping for each metabolite or a common damping for the major metabolites?
5. common damping for major metabolites except for Myo, because its frequency is near the water frequency?
6. two additional lipid/macromolecule signals below 1.8 ppm as additional support for the baseline?
7. the frequencies of glutamate and glutamine as independent fit parameters or the use of fixed frequencies in relation to the most prominent metabolite line of Naa?
8. relative amplitudes (referencing the metabolites to Cre3)?

## Results

The results using 11 pairs of acquisitions are summarized in Table 3. The protocol with the lowest *χ*^2^ is, as expected, No 5, the one using the most metabolites with freely adjustable parameters. In contrast to the lowest *χ*^2^ protocol 5 is not in the top category concerning the reproducibility.

**Fig. 4:**
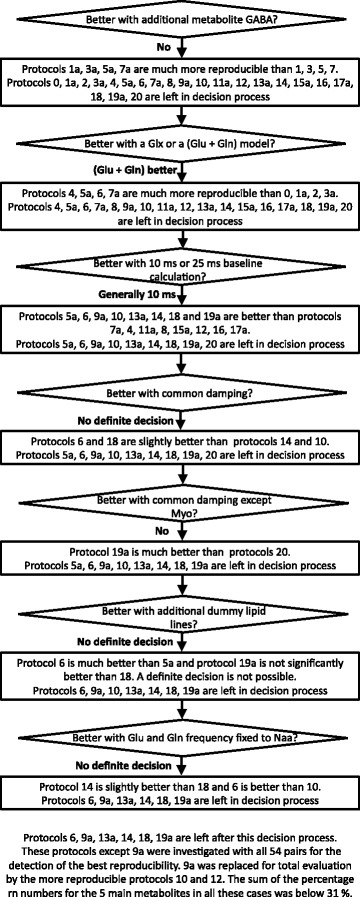
Decision flow chart showing the protocols tested on eleven pairs towards an increasing reproducibility

The flow chart of Fig. 4 shows the strategy for the evaluation of the best protocols using eleven pairs, one from each participant. For each question protocols with a significantly lower reproducibility performance were ruled out from further questions. Compared protocols with a difference in the sum of all rn numbers lower than 3 % were defined as similar and both were used for further investigations. Protocols 6, 9a, 13a, 14, 18, 19a are left after this decision process. These protocols except 9a were investigated with all 54 pairs for the detection of the best reproducibility. 9a was replaced for total evaluation by the more reproducible protocols 10 and 12. These seven protocols have the best reproducibility sum (< 31 %) of all main metabolite.

The protocols with the best results using all 54 pairs were protocols 14 and 18 (Table 4 and Fig. 3b).

Protocols to compare for each question are listed in the flow chart of Fig. 4. Concerning the prior defined questions the results are as follows.

1. *Is the reproducibility increased or decreased by the use of additional metabolites beside the clearly visible metabolites at 1.5 T?*
  Question 1 is investigated by comparing identical protocols differing only in the use of GABA. Protocols 1, 3, 5, 7 used GABA as an additional metabolite, whereas the protocols 1a, 3a, 5a, 7a excluded GABA. The protocols without GABA gave a better reproducibility as indicated by a smaller sum of the reproducibility numbers of the five major metabolites (see Fig. 4). Also the mean *χ*^2^ is, except for protocol five, less in the protocols without GABA.
2. *Is the reproducibility increased or decreased by the use of the sum of separately fitted glutamate and glutamine values instead of a predefined Glx model?*
  The comparison shows, that the use of the sum of separately fitted glutamate and glutamine values yields in all cases a much better reproducibility than in the six cases of a predefined Glx model.
3. *Is the reproducibility increased or decreased by the use of a “flat” or a more “curved” baseline using the first 10 ms or 25 ms for the base line calculation, respectively?*
  Using only the protocols not ruled out by question 1 and 2 the use of a more flat base line is generally better (protocols 5a, 6, 9a, 10, 13a, 14, 18 and 19a are better than protocols 7a, 4, 11a, 8, 15a, 12, 16, 17a).
4. *Is the reproducibility increased by the use of an independent damping for each metabolite or a common damping for the major metabolites?*
  On the basis protocol 6, 10, 14, 18 no decision is possible (protocols 6 and 18 are slightly better than protocols 14 and 10).
5. *Is the reproducibility increased by the use of a common damping for the major metabolites (see 4) except for Myo?*
  The use of a freely adjustable damping for Myo and a common damping for Naa, Cre3, Cho and Glx gave less reproducible results than the inclusion of Myo in the common damping (compare Table 3 protocol 20 and 19a).
6. *Is the reproducibility increased by the use of two additional lipid/macromolecule signals below 1.8 ppm as additional support for the baseline?*
  On the basis protocol 5a, 6, 18, 19a no decision is possible (protocol 6 is much better than 5a and protocol 19a is not significantly better than 18).
7. *Is the reproducibility increased or decreased by the use of the frequencies of glutamate and glutamine as independent fit parameters or the use of fixed frequencies in relation to the most prominent metabolite line of Naa?*
  On the basis protocol 6, 10, 14, 18 no definite decision possible. Protocol 14 is slightly better than 18 and 6 is better than 10.
8. *Is the reproducibility changed by the calculation of relative amplitudes (normalizing the metabolites using Cre3 as reference)?*
  The normalization is a division of two amplitudes with errors. So it is expected that the reproducibility is rather decreased by normalization. The largest decrease is found for Naa. For Cho and Glx the reproducibility is slightly decreased, whereas for Myo no relevant increase is found (see Table 4; 14, 18 vs. 14 ref, 18 ref). The reproducibility for normalized Naa, Cho and Glx was around 5 % whereas the reproducibility for Myo was close but above 9 % (Table 4).

Protocols 6, 9a, 13a, 14, 18, 19a are left after this decision process.

These remaining protocols except 9a were investigated with all 54 pairs for the detection of the best reproducibility. 9a was replaced for total evaluation by the more reproducible protocols 10 and 12. Summarizing the results of Table 4 using all 54 pairs, the protocols with most reproducible results for Naa, Cre3, Cho, Myo and Glx in lesion free white matter (protocols 14 and 18) have the following common features: no additional metabolites, Glx is taken as the sum of separately fitted Glu and Gln, a baseline modeled with the points of the first 10 ms, no additional lipid lines, common damping of all metabolites.

## Discussion

This investigation uses *in vivo* data for reproducibility assessment. An alternative would be the use of a set of theoretical metabolite bases with added statistical noise. The advantage of *in vivo* data from eleven individuals at different brain sides and different time points lays in the realistic concentration variation of the metabolite basis and the immanent use of non “visible” metabolites with a poor signal to noise ratio.

In contrast to other studies on reproducibility or variability [18–24] this investigation did not calculate the coefficient of variation for intra or inter subject reproducibility or variation depending on the location inside the brain. Instead two acquisitions with different noise and an identical metabolite basis are investigated for 54 pairs on their coincidence regarding the amplitudes of the main metabolites.

The protocols with the best results using all 54 pairs were protocols 14 and 18 (Table 4). Protocol 18 is favorable, mainly because protocol 18 achieves the same reproducibility as 14 with two parameters less: the frequencies of Glu and Glu were fixed in relation to the frequency of Naa. Additionally, in the reference version of protocol 18, 18_ref_, the reproducibility number rn of the pairs individually referenced to Cre3 as well as the sum of all rn’s were less for all cases except Cho in comparison to 14 ref.

The amplitudes of the metabolites strongly depend on the fitted baseline as also described by Hofmann et al. [25]. Depending on the curvature the values can differ significantly. In Fig. 3a for example the baseline had a broad peak between the lines of Cre2 and Cre3 and therefore the amplitude value (f. e.) for Cho is low in contrast to Fig. 3b with a more flat and lower baseline in this region.

This study did not vary all possible parameters. Additionally some parameters should have more steps. For example, using only the data of the first 10 or 25 ms for the base line were used; further investigations with additional times may improve the reproducibility. The default minimal signal to noise ratio for the amplitude of a metabolite was eight and never changed (see “Methods”). But every boundary in the signal to noise ratio–independent of the value–can impede the detection of a metabolite in only one of the two spectra building a pair. In such a case the normalized squared difference is large (four) assuming the not detected signal amplitude as zero (Eq. 1). This explains the large rn values for Glx in protocols 0 to 5a. Using protocols 0 to 3a (Table 5) in some cases the combined Glx was found only in one of the two spectra, whereas in case of protocol 4 to 5a this happened only for Gln with a lower signal to noise ratio compared to Glu, which was found in every case. A missing Gln in one of a pair increases the reproducibility number rn much less, than in the case of Glx.

**Table 5:** Reproducibility calculated as rn in percent for 54 pairs of consecutive spectra

Protocol	6	10	12	13a	14	*14 ref*	18	*18 ref*	19a
Naa	4.4	2.3	2.5	2.5	3.1	*4.5*	**2.2**	*4.2*	2.3
Cre3	4.9	4.7	4.3	4.5	4.4	*0*	**4.3**	*0*	4.4
Cho	5.3	5.3	4.7	4.7	**4.1**	*4.9*	4.8	*5.2*	4.9
Myo	12.9	23.7	10.0	11.3	9.3	*9.1*	**9.0**	*9.0*	9.7
Glx	7.7	5.7	12.9	13.5	**4.6**	*6.0*	5.2	*5.7*	5.5
Sum of all met.	35.1	41.8	34.4	36.4	**25.5**	*24.5*	**25.5**	*24.2*	26.8
Naa, Cre3, Cho	14.5	12.4	11.5	11.7	11.6	*(9.4)*	**11.3**	*(9.4)*	11.6
all–Myo	22.2	18.2	24.4	25.2	**16.2**	*15.4*	16.5	*15.1*	17.1
all–Glx	27.4	36.1	21.5	23.0	20.9	*18.5*	**20.3**	*18.5*	21.3
amp.(Naa)	190.8	206.9	181.0	180.4	200.0	*1.9*	208.2	*2.0*	209.0
amp.(Cre3)	96.5	95.9	97.3	97.5	104.5	*1*	105.4	*1*	106.5
amp.(Cho)	28.2	28.5	29.4	29.3	31.5	*0.3*	31.7	*0.3*	32.0
amp. (Myo)	55.3	66.9	57.1	57.9	68.4	*0.6*	71.5	*0.7*	71.4
amp. (Glx)	226.5	279.7	155.9	159.7	322.3	*3.1*	261.8	*2.5*	255.7
±% of mean									
Naa	13.1	**6.1**	6.4	6.3	8.01	*12.8*	6.3	*11.1*	6.3
Cre3	11.2	13.2	11.7	11.5	**10.9**	*0.0*	11.4	*0.0*	11.1
Cho	11.9	13.8	13.8	14.4	**10.0**	*15.2*	13.9	*18.3*	13.3
Myo	57.2	88.2	35.7	50.2	34.0	*33.4*	**28.2**	*28.2*	28.9
Glx	21.2	16.2	29.2	30.8	**11.9**	*14.9*	13.3	*12.0*	13.5

The minimal values for each of the five main metabolites are marked bold. The values of the referenced metabolites are in italic. Protocol 18 shows up together with protocol 14 the best reproducibility rated by the sum of the five rn’s of the main metabolites. Protocol 18 is preferable, because

1. it needs less fit parameters for identical reproducibility results

2. the rn sum of the major metabolites is minimal for 18 ref, because rn of Cre3 is minimal in protocol 18

Note: In all cases the rn calculated with 54 pairs is similar as in the case of 11 pairs (compare Table 3 with Table 4). This legitimates the missing calculations with all 54 pairs for the protocols not enlisted in Table 4

“ref” indicates that the amplitude of a metabolite is referenced to Cre3, which is the calculation of relative concentrations *i.e.* evaluatedas amp_ref_(met) = amp(met)/amp(Cre3)

“±% of mean” for each metabolite (five last lines) is the maximal percentage deviation from the mean value for a pair of amplitudes

The residual water suppression in this study was implemented as an iterative filter with 22 stages. The experience of the user (MB) preferred this value instead of the default value of 10. No other stage numbers have been tested, because recent post processing software algorithms (jMRUI [11, 26–29] or Syngo Via (Siemens Healthcare)) prefer instead of an iterative filter a singular value decomposition (SVD) algorithm for residual water suppression. This was not possible with the work in progress software metabolite report, but both methods are available in the final product Syngo Via. The comparison of SVD and iterative filter within Syngo Via is not done yet.

This study did not examine absolute metabolite concentrations. Only the amplitudes or relative amplitudes with Cre3 as reference (amp_met_/amp_Cre3_) of directly comparable pairs were evaluated. The reason on one hand is the intention to search for an optimized reproducibility not needing a reference with a known concentration. On the other hand the reason is the lack of consensus for acquisition and post-processing methods to evaluate absolute metabolite concentration. Published results show “considerable variations in the approaches” [8] and therefore a large span for absolute concentrations. For the metabolite Naa with the best reproducibility, the concentration inside white matter in healthy subjects can differ from 9.7 to 18.4 mmol/l [30–34]. The values depend on sequence design, acquisition system, post-processing algorithm as well as post-processing parameters, relaxation corrections, internal or external references or subject gender, subject age and the position inside the brain [24].

All spectra of this investigation were acquired in “lesion free” (no lesion signs found in MR imaging) white matter brain tissue. Spectra of brain lesions, for example tumors, can be drastically different. For example the lipid components in/around high-grade brain tumors normally are extremely large compared to the small (often zero) values found in healthy tissue. In our examination the detected lipids were very small and superimposed or dominated by macromolecules. In most of our pairs no or only one lipid line was found as expected for healthy subjects without a sign for any brain disease. For that reason the application of the post processing parameters of this investigation is limited to lesion free tissue. But despite of this limitation the application to studies is broad, because alterations in brain biochemistry of lesion free areas had been detected nearby tumors [35–37], in patients with multiple sclerosis [38], drug abuse [39–41], AIDS [42, 43] or psychiatric disorders [44].

## Conclusion

The prerequisites of this study were optimal for *in vivo* comparisons. The brain is one of the organs that can be easily fixed inside the scanner. Spectrum pairs were acquired within a few minutes guaranteeing a stable metabolite basis for comparisons. All pairs were similar, because only lesion free white matter of healthy subjects at an identical brain position was investigated.

Metabolite amplitudes inside lesion free healthy brain can be quantified with a calculated accuracy (rn(met)) around 5 % for Naa, Cho and Glx using Cre3 as reference. The reproducibility of Myo is roughly twice as bad. Assuming these rn, the real metabolite amplitudes calculated with these post-processing parameters are most likely within a twofold interval of ±10 or ±20 % respectively. The reproducibility (rn) should be similar using other references like internal or external water for an absolute concentration evaluation and are not influenced by relaxation corrections with literature values, which is only a multiplication by a constant factor.
